# The Shozu Herpes Zoster (SHEZ) Study: Rationale, Design, and Description of a Prospective Cohort Study

**DOI:** 10.2188/jea.JE20110035

**Published:** 2012-03-05

**Authors:** Yukiko Takao, Yoshiyuki Miyazaki, Fumitake Onishi, Hideaki Kumihashi, Yasuyuki Gomi, Toyokazu Ishikawa, Yoshinobu Okuno, Yasuko Mori, Hideo Asada, Koichi Yamanishi, Hiroyasu Iso

**Affiliations:** 1 The Research Foundation for Microbial Diseases of Osaka University, Kagawa, Japan; 2 Public Health, Department of Social and Environmental Medicine, Osaka University, Graduate School of Medicine, Osaka Japan; 3 National Institute of Biomedical Innovation, Osaka, Japan; 4 Nara Medical University School of Medicine, Nara Japan

**Keywords:** herpes zoster, skin test, incidence, prospective cohort study, cell-mediated immunity

## Abstract

**Background:**

The incidence and risk factors for herpes zoster have been studied in cross-sectional and cohort studies, although most such studies have been conducted in Western countries. Evidence from Asian populations is limited, and no cohort study has been conducted in Asia. We are conducting a 3-year prospective cohort study in Shozu County in Kagawa Prefecture, Japan to determine the incidence and predictive and immunologic factors for herpes zoster among Japanese.

**Methods:**

The participants are followed for 3 years, and a telephone survey is conducted every 4 weeks. The participants were assigned to 1 of 3 studies. Participants in study A gave information on past history of herpes zoster and completed health questionnaires. Study B participants additionally underwent varicella-zoster virus (VZV) skin testing, and study C participants additionally underwent blood testing. If the participants develop herpes zoster, we evaluate clinical symptoms, measure cell-mediated immunity and humoral immunity using venous blood sampling, photograph skin areas with rash, conduct virus identification testing by polymerase chain reaction (PCR) and virus isolation from crust sampling, and evaluate postherpetic pain.

**Results:**

We recruited 12 522 participants aged 50 years or older in Shozu County from December 2009 through November 2010. The participation rate was 65.7% of the target population.

**Conclusions:**

The present study is likely to provide valuable data on the incidence and predictive and immunologic factors for herpes zoster in a defined community-based population of Japanese.

## INTRODUCTION

Varicella-zoster virus (VZV) causes varicella in childhood in the initial stage of infection.^1^^,^^2^ After initial infection, VZV is believed to latently infect the sensory nerve ganglia throughout the lifetime of the host and is reactivated by immunocompromise, aging, stress, overwork, and a decline in cell-mediated immunity, which leads to herpes zoster.^3^ Epidemiologic studies of herpes zoster have examined incidence rate,^4^^–^^7^ age^4^^–^^13^ and sex^4^^,^^6^^–^^10^^,^^14^^,^^15^ differences, seasonality,^6^^,^^7^^,^^9^^,^^11^^,^^16^^,^^17^ family history,^18^^,^^19^ underlying diseases,^15^^,^^20^^–^^24^ lifestyle factors,^25^^,^^26^ and sociopsychological factors.^6^^,^^26^^,^^27^

The Oka/Merck VZV vaccine has been used in the United States and is at least 14 times more potent than the Oka/Merck Varivax vaccine. The VZV vaccine was shown to have a preventive effect and was approved as a herpes zoster vaccine.^4^ The Advisory Committee for Immunization Practices (ACIP) now recommends its use for adults aged 60 years or older.^7^

In Japan, there has been no epidemiologic study of herpes zoster. Thus, its epidemiology remains unclear and no herpes zoster vaccine has been developed in this country.

We are conducting a 3-year prospective cohort study in Shozu County, Kagawa Prefecture to determine the incidence and predictive and immunologic factors for herpes zoster. Herpes zoster is believed to occur when cell-mediated immunity declines,^28^^,^^29^ but there are no data regarding the threshold level of cell-mediated immunity for clinical occurrence. Therefore, in this study, we are investigating the relationship between cell-mediated immunity, as evaluated by the VZV skin test (using the Varicella antigen Biken),^30^^–^^32^ and risk of herpes zoster. Further, the measurement of cell-mediated and humoral immunity was conducted to assess their relation with the result of the VZV skin test. If the skin test proves useful as a predictor of herpes zoster, individuals at high risk can be identified. The skin test might also be useful as an index of vaccine effectiveness.

## METHODS

### Research community

Shozu County, Kagawa Prefecture is an administrative division consisting mainly of Shodoshima Island (area: 153.30 km^2^) and Teshima Island (area: 14.49 km^2^) (Figure). On 1 July 2008, it had a census population of 33 782, of which 32.8% were aged 65 years or older.

**Figure.**
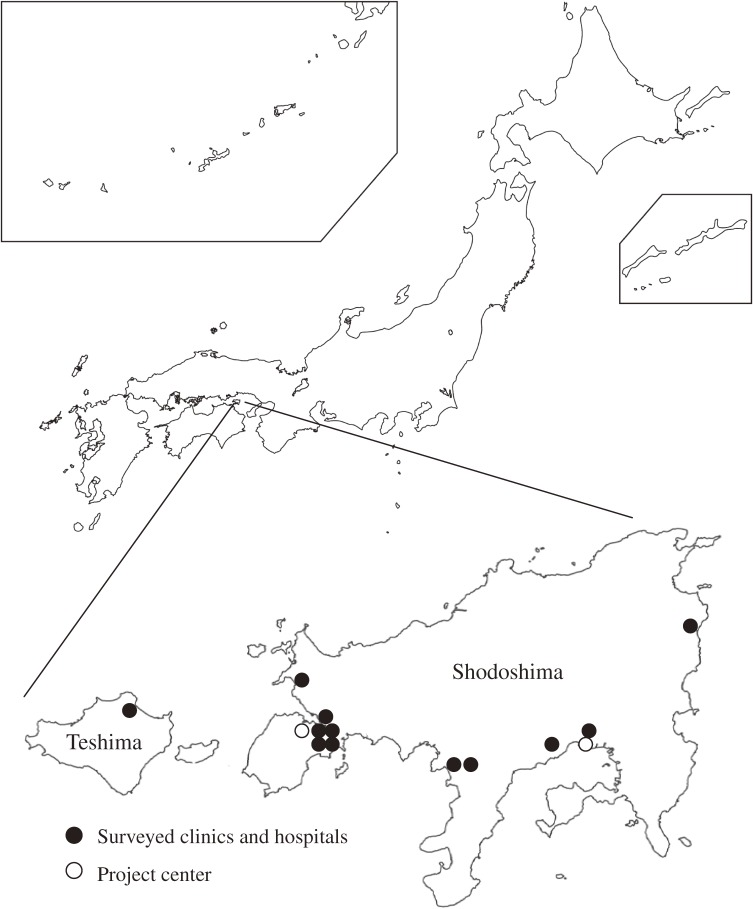
Locations of Shozu County in Shodoshima and Teshima Islands, surveyed clinics and hospitals, and project center

This research area was selected according to the following 4 criteria: (1) population stability, (2) availability of self-government bodies that can smoothly disseminate information to the population, which leads to a high registration rate, (3) good cooperation by island clinics and hospitals (where most residents seek health consultations), which facilitates collection of information on the occurrence of herpes zoster in the cohort, and (4) presence of a field research team that can collect biological samples (blood, crust, or bulla fluid) from individuals who develop herpes zoster and rapidly transport the samples to the central laboratory.

### Target population and participants

The target population was Japanese residents of Shozu County aged 50 years or older on 1 October 2008. Although herpes zoster can occur at any age, incidence rapidly increases after age 50 years.^10^^,^^13^ Thus, the target population was restricted to those aged 50 years or older.

A provisional participant was defined as a person who signed the registration form. The items on the registration form were address, telephone number, name, sex, date of birth, age, the participant’s choice of study category, the clinics or hospitals that the participant would probably consult if herpes zoster occurred, and the most convenient time of day for telephone surveys. The documents necessary to complete registration were sent to 12 896 provisional participants by confidential mail, and those who completed the procedure were formally registered. Ultimately, there were 12 522 final participants.

### Study period

The duration of the survey is 3 years after registration, ie, between April 2009 and November 2012.

### Study category

As shown in Table 1, there are 3 studies: A, B, and C. For study A, we conducted a questionnaire and telephone survey, and, if the participant develops herpes zoster during the study period, surveys are completed at onset and during recovery (the basic survey). In addition to the basic survey, study B participants underwent VZV skin testing at registration to examine cell-mediated immunity while healthy. Study C assesses cell-mediated immunity (ELISPOT) and humoral immunity (neutralizing antibody, gp-ELISA, and IAHA) using serum collected at registration and 1, 2, and 3 years later among participants selected from study B.

**Table 1.** Study framework

		Timeline			
		
		Baseline	1 year	2 years	3 years
Study A	(No. = 12 000)				
	Health questionnaire	×	NA	NA	NA
	Telephone survey^a^	×	×	×	×
			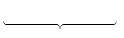		
	Surveys and tests at ​ onset and recovery		Onset	3 months	
		
	Clinical questionnaire		×	×	
	Blood immunologic ​ testing		×	×	
	PCR/viral isolation		×	NA	

Study B	(No. = 5000 to 7500)				
	VZV skin testing	×	NA	NA	NA

Study C	(No. = 200 to 300)				
	VZV skin testing	×	×	×	×
	Blood immunologic ​ testing	×	×	×	×

^a^Conducted every 4 weeks.

Target enrollment number is shown in parentheses.

NA: not available.

The participants chose 1 of the 3 studies, with restrictions on the number of entries, ie, 5000 to 7500 for study B and 200 to 300 for study C. The participants in study C were selected randomly by drawing lots because the participants who desired enrollment exceeded the target numbers. Ultimately, 5685 and 365 of the 12 522 participants were enrolled in studies B and C, respectively.

### Study hypotheses

We are testing the following major hypotheses in study A (Table 2): (1) the annual age-adjusted incidence of herpes zoster is approximately 10 per 1000 persons aged 50 years or older,^4^ (2) the incidences of herpes zoster and postherpetic neuralgia increase with age,^4^^,^^5^ (3) there is no sex difference in the incidences of herpes zoster and postherpetic neuralgia,^4^^,^^8^^,^^10^ (4) the incidence of herpes zoster is highest from July through October, which is the period of minimum incidence for chicken pox,^9^^,^^11^^,^^12^ and there is no seasonality in postherpetic neuralgia, (5) underlying diseases, stress, sleep deprivation, inadequate intake of fruits and vegetables, smoking, drinking, depression, and family history of herpes zoster increase the risks of herpes zoster and postherpetic neuralgia,^15^^,^^19^^–^^26^ (6) perceived good health, exercise, optimism, positive well-being, and social support are associated with enhanced reaction on the VZV test and decreased risks of herpes zoster and postherpetic neuralgia,^26^^,^^27^ and (7) greater pain at onset of herpes zoster is associated with increased risk of postherpetic neuralgia.

**Table 2.** Study category and hypotheses

Study category	Hypotheses	
Study A	1.	The annual age-adjusted incidence of herpes zoster is approximately 10 per 1000 persons aged ≥50 years.
	2.	The incidences of herpes zoster and postherpetic neuralgia increase with age.
	3.	There is no sex difference in the incidences of herpes zoster and postherpetic neuralgia.
	4.	The incidence of herpes zoster is highest from July to October, which is the period of low incidence for chicken pox; there is no seasonality in postherpetic neuralgia.
	5.	Underlying diseases, stress, sleep deprivation, inadequate intake of fruits and vegetables, smoking, drinking, depression, and family history of herpes zoster all increase the risks of herpes zoster and postherpetic neuralgia.
	6.	Perceived good health, exercise, optimism, positive well-being, and social support are associated with enhanced reactivity to the VZV test and decreased risks of herpes zoster and postherpetic neuralgia.
	7.	Pain at onset of herpes zoster is positively associated with the risk of postherpetic neuralgia.

Study B	1.	The state of cell-mediated immunity against herpes zoster can be evaluated with the VZV skin test.
	2.	Reactivity to the VZV skin test is inversely associated with the risk and severity of herpes zoster and postherpetic neuralgia.

Study C	1.	Cell-mediated and humoral immunity decline with age.
	2.	Without the boosting effect of exposure to varicella-zoster virus, eg, due to an epidemic of varicella, cell-mediated and humoral immunity gradually decline.

The hypotheses in study B are (1) the state of cell-mediated immunity against herpes zoster can be evaluated by the VZV skin test,^30^^–^^32^ and (2) the stronger the response to the VZV skin test, the lower the risk and severity of herpes zoster and postherpetic neuralgia.^29^ The hypotheses in study C are (1) levels of cell-mediated and humoral immunity decline with age,^32^ and (2) without the boosting effect caused by exposure to varicella-zoster virus, eg, during an epidemic of varicella, levels of cell-mediated and humoral immunity gradually decline.^16^

### Interview regarding past history

The registration interview was conducted by research physicians to identify a past history of herpes zoster. The question items asked whether the participant had ever developed herpes zoster and, if so, when did it occur and did they see a medical doctor. Responses were obtained from all 12 522 final participants (100%).

In studies B and C, to increase the reliability of the past history, research physicians asked participants to describe the symptoms they experienced and the drugs administered and determined whether the past event in question was indeed herpes zoster.

### Self-administered health questionnaires

A self-administered questionnaire survey was administered at registration to evaluate other factors, including diet, stress and overwork. If an answer was inadequate, secretariat members confirmed the information by asking the participants.

The question items inquired about self-rated health, smoking, exercise, walking time, sleep, degree of satisfaction with sleep, stress, diet (frequencies of fruits, vegetables, fish, meat, eggs, miso soup, soybeans, and milk intakes), drinking, personality, frequency of laughing, purpose of life, opportunities for conversation, spiritual support, life events, hobbies and interests, hopes, underlying diseases, family history, height, and body weight. Valid answers were obtained from 12 360 (98.7%) of the 12 522 final participants.

### Telephone survey

A contract organization (Bellsystem24 Inc.) was commissioned to conduct the telephone surveys, which are performed according to an established script at an interval of 4 weeks during the study. All participants are asked whether they developed symptoms suggestive of herpes zoster. One participant in each family answers the surveys for all participants in the family.

The questions ask about the presence or absence of rash, pain, history of contact with a varicella patient, and admission to a clinic or hospital. If a participant reports both rash and pain, this information is automatically transmitted to the secretariat, and the survey on onset is given.

### VZV skin test

We use the varicella antigen Biken (The Research Foundation for Microbial Diseases of Osaka University) to evaluate the sensitivity or status of cell-mediated immunity. The test is performed by intradermal inoculation of varicella antigen Biken at 0.1 mL/injection and inspection of the inoculation site after 48 hours. The VZV skin test was performed at registration for the 5685 participants enrolled in study B.

### Blood tests

Blood tests were performed for all participants enrolled in study C. Cell-mediated immunity (ELISPOT) and humoral immunity (neutralizing antibody, gp-ELISA, and IAHA) are examined at baseline and after 1, 2, and 3 years.

### Surveys at onset and recovery

A survey at onset is given if a participating physician diagnoses herpes zoster or possible herpes zoster. The survey at onset comprises 5 items: (1) evaluation of clinical symptoms, (2) measurement of cell-mediated and humoral immunity using venous blood sampling, (3) virus identification testing by PCR and virus isolation from crust sampling, (4) evaluation of pain, and (5) photographing skin areas with rash. The survey during recovery comprises 2 items: evaluation of sequelae and measurement of cell-mediated and humoral immunity using venous blood sampling.

A survey form is used in physician evaluations of the severity of clinical symptoms and treatment. The evaluation items are presence or absence of underlying diseases, use of an immunosuppressant or antineoplastic agent, date of rash appearance, distribution of rash, rash properties (presence or absence of erythema; numbers of vesicles, pustules, and erosions; and presence or absence of ulceration, fusion, and bloody vesicles), date of pain onset, other specific symptoms (fever, headache, generalized herpes zoster, multidermatomal herpes zoster, eye complications, motor paralysis, Ramsay Hunt syndrome and others), and treatment.

Evaluation of onset and postherpetic pain is conducted by secretariat members using a modified Zoster Brief Pain Inventory survey form.^33^ The question items are presence or absence of stress and pain, distribution of pain, severity of pain according to a face scale from 0 (no pain) to 5 (sleep disturbing pain), changes in tactile sensation in painful areas, treatment or medication for pain, and quality of life (activities of daily living, psychology, work and housework, social events, sleep, and hobbies).

Questionnaires are scheduled on days 0, 1, 2, 3, 4, 5, 6, 13, 20, 27, 34, 41, 48, 55, 85, 115, 145, and 175 after the initial examination. The questionnaires finish with the disappearance of pain but are continued for at least 7 days. Also, the presence of mental stress was evaluated at the initial examination only, and treatment or medication for pain is evaluated at the initial, weekly, and monthly examinations.

The survey during recovery is conducted by a physician using a survey form that asks about the presence of sequelae (postherpetic neuralgia, motor paralysis, scars, and others).

### Definitive diagnosis of herpes zoster

All participants with a new clinical diagnosis of either herpes zoster or possible herpes zoster are examined by PCR analysis for VZV, HSV, and beta-globin DNA in fluid from vesicles. In addition, they undergo serologic analysis for anti-VZV antibodies using paired serum and have clinical photographs taken. After a comprehensive evaluation of clinical symptoms and the results of PCR and serologic tests, the final diagnosis is determined by the clinical evaluation committee, which consists of 3 dermatologists from Nara Medical University School of Medicine who have expertise in herpes zoster.

If the PCR assay reveals VZV DNA, the final diagnosis is herpes zoster; if the assay is positive for beta-globin or HSV DNA and negative for VZV DNA, the final diagnosis is not herpes zoster. If the specimen obtained for PCR assay is inadequate (ie, negative for both viral and beta-globin DNA) or missing, the final diagnosis is determined by the clinical evaluation committee on the basis of serologic testing and clinical symptoms. Participants with a final diagnosis of herpes zoster are regarded as definitively diagnosed patients.

### Dropouts

Participants are regarded as drop outs when continuation of the survey is judged to be impossible due to participant death, impaired cognitive function, departure from the survey area, or if the participant gives notice of withdrawal or does not respond after 3 or more telephone survey attempts. Death of participants is confirmed with the cooperation of the municipal administration, using participant name and date of birth.

### Sample size calculation

Among the census population of 19 138 (as of 1 June 2007), at least 12 000 (≥62.7%) were expected to be registered for study A (Table 1). The incidence of herpes zoster is calculated separately for each sex and age category (50–59, 60–69, and ≥70 years). In previous studies in Japan and abroad, the annual incidence of herpes zoster was reported to be 0.5% to 0.6% for persons aged 50 to 59 years, 0.6% to 1.0% for those aged 60 to 69 years, and 0.9% for those aged 70 years or older, with no difference by sex.^4^^,^^5^^,^^9^

When the expected numbers of patients during the 3-year study period are calculated by multiplying the median value of incidence in each age category (0.55% for persons aged 50–59 years, 0.80% for those aged 60–69 years, and 0.90% for persons aged ≥70 years) by the census populations of the respective age category in Shozu County (Table 1), herpes zoster is expected to develop in 48 men and 47 women aged 50 to 59 years, 53 men and 59 women aged 60 to 69 years, and 90 men and 146 women aged 70 years or older.

To calculate sex- and age-specific incidence with sufficient reliability, at least 30 patients must develop herpes zoster during the 3-year survey period in each sex/age category. The population of Shozu County that is aged 50 years or older was 19 138, and approximately 12 000 (63%) was therefore set as the target number of participants. Under these conditions, a difference in incidence during the 3-year survey period between men aged 50 to 59 years (*n* = 2891) and those aged 70 years or older (*n* = 3323) can be detected at a significance level of 5% and a statistical power of 80%.

To examine the relationship between the VZV skin test and the risk of herpes zoster for study B, the skin test result is classified into tertiles, and the 3-year incidence in the high-reaction group (highest tertile) is assumed to be 1.2% (annual incidence: 0.4%). When the risk of herpes zoster in 1600 (up to a maximum of 2500) participants in the high-reaction group relative to that in the 1600 (up to a maximum of 2500) participants in the low-reaction group (lower 33 percentiles) is calculated, the association between the VZV skin test result and the risk of herpes zoster can be detected if the relative risk is 2.1 or greater (<1600 participants in each tertile), or 1.9 or greater (<2500 participants in each tertile), at a significance level of 5%, analytical power of 80%, and follow-up rate of 90%. Thus, we aimed to obtain 5000 to 7500 participants for study B (Table 1).

To evaluate the duration of cell-mediated and humoral immunity in study C, the state of immunity is classified into tertiles, and analysis is performed separately by sex. To ensure enrollment of 30 or more participants in each tertile group for each sex, the target enrollment number set at 200 to 300.

### Ethical issues

Informed consent for the study was obtained from participants in study B and study C. For study A participants, the return of questionnaires was regarded as consent to participate in the study.

The present study was approved by the Ethics Committee of the Research Foundation for Microbial Diseases of Osaka University, Osaka University Graduate School of Medicine, National Institute of Biomedical Innovation, and Nara Medical University School of Medicine.

## RESULTS

The census population aged 50 years or order was 19 058 individuals (8424 men and 10 634 women in total), consisting of 5362 (2710 and 2652) adults aged 50 to 59 years, 4918 (2383 and 2535) aged 60 to 69 years, 4897 (2064 and 2833) aged 70 to 79 years, and 3881 (1267 and 2614) aged 80 years or older.

Table 3 shows the number of participants and their age distribution and past history of herpes zoster. We successfully recruited 12 522 participants (65.7% of all residents) for study A, 5685 participants for study B, and 365 participants for study C. When the target population was restricted to residents aged 50 years or older who actually lived in Shozu County (*n* = 17 323), the participation rate for study A reached 72.3%. As compared with the census population, the participants in study A had a similar mean age, but those in study B had a slightly lower mean (*P* < 0.001). In study A, the prevalence of a self-reported past history of herpes zoster was 12.7% for men and 20.1% for women (*P* for sex difference <0.001).

**Table 3.** Baseline characteristics of participants in studies A, B, and C

	Men	Women	Total
Number of census population	8424 (44.2)	10 634 (55.8)	19 058
Age, years			
Mean ± SD	66.7 ± 10.8	70.0 ± 11.9	68.6 ± 11.6
50–59	2710 (32.2)	2652 (24.9)	5362 (28.1)
60–69	2383 (28.3)	2535 (23.8)	4918 (25.8)
70–79	2064 (24.5)	2833 (26.6)	4897 (25.7)
≥80	1267 (15.0)	2614 (24.6)	3881 (20.4)

**Study A**			
Number of participants	5589 (44.6)	6933 (55.4)	12 522
Age, years			
Mean ± SD	67.2 ± 10.1	68.9 ± 10.9	68.2 ± 10.6
50–59	1505 (26.9)	1685 (24.3)	3190 (25.5)
60–69	1836 (32.9)	1976 (28.5)	3812 (30.4)
70–79	1458 (26.1)	1948 (28.1)	3406 (27.2)
≥80	788 (14.1)	1326 (19.1)	2114 (16.9)
Self-reported history of ​ herpes zoster, Yes	717 (12.8)	1394 (20.1)	2111 (16.9)

**Study B**			
Number of participants	2574 (45.3)	3111 (54.7)	5685
Age, years			
Mean ± SD	66.4 ± 9.2	67.0 ± 9.6	66.7 ± 9.5
50–59	693 (26.9)	839 (27.0)	1532 (27.0)
60–69	942 (36.6)	1015 (32.6)	1957 (34.4)
70–79	675 (26.2)	903 (29.0)	1578 (27.8)
≥80	264 (10.3)	354 (11.4)	618 (10.9)
Self-reported history of ​ herpes zoster, Yes	423 (16.4)	707 (22.7)	1130 (19.9)
Confirmed past history of ​ herpes zoster, Yes	384 (14.9)	663 (21.3)	1047 (18.4)

**Study C**			
Number of subjects	181 (49.6)	184 (50.4)	365
Age, years			
Mean ± SD	73.5 ± 7.8	73.9 ± 7.6	73.7 ± 7.7
50–59	—	—	—
60–69	60 (33.2)	61 (33.2)	121 (33.2)
70–79	61 (33.7)	64 (34.8)	125 (34.3)
≥80	60 (33.2)	59 (32.1)	119 (32.6)
Self-reported history of ​ herpes zoster, Yes	35 (19.3)	59 (32.1)	94 (25.8)
Confirmed past history of ​ herpes zoster, Yes	34 (18.8)	56 (30.4)	90 (24.7)

Percentages are shown in parentheses.

A similar sex difference was found in each age group when the participants were stratified by age group (Table 4).

**Table 4.** Number (prevalence) of participants with a self-reported past history of herpes zoster by sex and age group

Age, years	Men	Women	*P* for sex difference
50–59	139 (9.2)	231 (13.7)	<0.001
60–69	219 (11.9)	423 (21.4)	<0.001
70–79	217 (14.9)	446 (22.9)	<0.001
≥80	142 (18.0)	294 (22.2)	0.02
Total	717 (12.8)	1394 (20.1)	<0.001

## DISCUSSION

We successfully recruited a total of 12 522 residents of Shozu County aged 50 years or older, for a participation rate of 65.7% of the census population and 72.3% of residents who actually lived in Shozu County. To ensure high follow-up and complete ascertainment of the occurrence of herpes zoster, a systematic telephone survey is underway. The residents are informed of the study by notices on bulletin boards and by brochures sent every 3 months to enhance resident interest, increase knowledge of herpes zoster, and encourage early consultation at medical centers. Early consultation is expected to contribute to rapid administration of antiviral agents, which should reduce the severity of herpes zoster and postherpetic neuralgia. In addition, increased knowledge of herpes zoster among residents of the survey community might encourage residents with mild herpes zoster to consult a physician at a local hospital or clinic, which should also increase the accuracy of incidence estimates.

The occurrence of herpes zoster is reported to be affected by varicella epidemics.^11^^,^^16^ Because such epidemics are associated with vaccination status, varicella epidemics and vaccination status are monitored in the survey community through the *Infectious Diseases Weekly Report Kagawa*, the annual sales report for the Biken varicella vaccine, and the population census.

In Shozu County, the numbers of varicella symptoms observed varied between 2001 and 2010. The average of the numbers reported from 2 sentinel pediatrics clinics in the area during that 10-year period was 113.5 people (range, 22–266), and epidemics (≥150 cases) occurred in 2002, 2004, and 2010 according to the *Infectious Diseases Weekly Report Kagawa*. The proportion of individuals vaccinated against varicella was 51.4% (amount of vaccine sold in 2008 divided by the number of childbirths in 2007). This proportion was 9.8% higher than the Japan national average, according to the annual sales report for the Biken varicella vaccine and population census. Therefore, it is necessary to continue to investigate vaccination status and outbreaks and to verify their relationship with herpes zoster incidence.

The present study is expected to provide valuable data on incidence and predictive and immunologic factors for herpes zoster in a defined, community-based Japanese population.
